# Unveiling the heritability of selected unexplored pharmacogenetic markers in the Saudi population

**DOI:** 10.3389/fphar.2025.1559399

**Published:** 2025-05-01

**Authors:** Mohammad A. Alshabeeb, Deemah Alwadaani, Sireen A. R. Shilbayeh, Fatemah A. Alherz, Ali Alghubayshi

**Affiliations:** ^1^ Pharmaceutical Analysis Department, King Abdullah International Medical Research Center (KAIMRC), Riyadh, Saudi Arabia; ^2^ King Saud Bin Abdulaziz University for Health Sciences (KSAU-HS), Ministry of National Guard Health Affairs (MNGHA), Riyadh, Saudi Arabia; ^3^ Medical Genomics Research Department, KAIMRC, Riyadh, Saudi Arabia; ^4^ Department of Pharmacy Practice, College of Pharmacy, Princess Nourah bint Abdulrahman University, Riyadh, Saudi Arabia; ^5^ Department of Pharmaceutical Sciences, College of Pharmacy, Princess Nourah bint Abdulrahman University, Riyadh, Saudi Arabia; ^6^ Clinical Pharmacy Department, University of Hail, Hail, Saudi Arabia; ^7^ Department of Pharmacotherapy and Outcomes Science, School of Pharmacy, Virginia Commonwealth University, Richmond, VA, United States

**Keywords:** Pharmacogenomics (PGx), ATIC, CHRNA5, CYP3A4, IFNL3, SLC19A1, SLCO1B1, Saudis

## Abstract

**Background:**

Pharmacogenomic (PGx) variants can significantly impact drug response, but limited data exists on their prevalence in Middle Eastern populations. This study aimed to investigate the inheritance of certain markers in candidate pharmacogenes among healthy Saudis.

**Methods:**

DNA samples from 95 unrelated healthy Saudi participants were genotyped using the Affymetrix Axiom Precision Medicine Diversity Array. Thirty-eight variants in 15 pharmacogenes were analyzed based on their clinical relevance and lack of previous reporting in Saudi populations.

**Results:**

Twenty-six of the 37 tested markers were undetected in the cohort. The selected variants in six genes [*DPYD* (rs1801268), *CACNA1S* (rs772226819), *EGFR* (rs121434568), *RYR1* (rs193922816), *CYP2B6* (rs3826711), and *MT-RNR1* (rs267606617, rs267606618, rs267606619)] were found to be non-existing among Saudis. In contrast, 11 variants and alleles in nine pharmacogenes were detected at varying frequencies. Notable findings included high frequencies of variants in *ATIC* [rs4673993, minor allele frequency (MAF) = 0.71)] and *SLC19A1* (rs1051266, MAF = 0.48) affecting methotrexate efficacy. Three alleles were identified in *CYP3A4*, including a common (*CYP3A4* rs2242480) and two rare alleles (*3 and *22). Another three markers [rs16969968 in *CHRNA5*, rs11881222 in *IFNL3* (*IL28B*), and SLCO1B1*14] were found to be highly distributed among the participants (MAF = 0.35, 0.30, and 0.14, respectively). Conversely, three rare markers: CYP2A6*2, NAT2*14, and rs115545701 in *CFTR*, were identified at low-frequency levels (MAF = 0.021, 0.011, 0.005, respectively). Statistically significant differences in allele frequencies were observed for eight variants between Saudi and African populations, five variants compared to East Asians, and two variants compared to Europeans.

**Conclusion:**

This study provides novel insights into the distribution of clinically relevant PGx variants in the Saudi population. The findings have implications for personalizing treatments for various conditions, including rheumatoid arthritis, cystic fibrosis, and hepatitis C. These data contribute to the development of population-specific PGx testing panels and treatment guidelines.

## 1 Introduction

An individual’s genetic composition can affect their pharmacological responses, including variations in drug metabolism, therapeutic efficacy, and susceptibility to adverse drug reactions (ADRs) (Pirmohamed, 2023). Different populations can have unique genetic variations that influence drug response (Biswas et al., 2023). Over the past 2 decades, a large number of studies have been published describing the pharmacogenomic (PGx) variants and their allele frequencies among different populations, particularly in European, American, African, and Southeast Asian nations (e.g., Japanese and Chinese). While Europe and the United States have well-established research infrastructures (van der Wouden et al., 2017; McDermott et al., 2022), very limited PGx research has been performed to identify ancestral diversity and differences in variants inheritance across Middle Eastern populations (Jithesh et al., 2022; Abou Tayoun et al., 2021). Understanding the prevalence and distribution of PGx variants among different populations is crucial for precision medicine practice. Thus, testing of these variants can ensure patients receive optimal treatment based on their genetic profile (Sarwar, 2023). Several cost-effectiveness studies have shown that genetic testing can help reduce healthcare expenditure by minimizing the costs related to preventable ADRs and treatment failures (Angamo et al., 2016; Brixner et al., 2016; Deenen et al., 2016).

Few reports have investigated the carriage of common and rare variants in 35 pharmacogenes among the Saudi population (Mizzi et al., 2016; Goljan et al., 2022; Alshabeeb et al., 2022b). The common risk alleles were noticed in several genes encoding cytochrome P450 enzymes such as *CYP2D6* (*2, *4,*10, and *41), *CYP2C9* (*2 and *3), *CYP2C19* (*2 and *17), *CYP3A5* (*3), *CYP4F2* (*3). Other common mutations in different genes [e.g., *ABCG2* (rs2231142), *ACE* (rs1799752), *ADD1* (rs4961), *ADRB2* (rs1042713), *APOE* (rs7412), *HLA-A* (*31:01), and *HLA-C* (*04:01)] were also detected. In contrast, multiple rare mutations were reported in various genetic loci, for example, *CES1* (rs71647871), *CYP2D6* (*5, *6, *17, and *29), CYP3A5*3, *DPYD* (1236G>A), *TPMT* (*3A and *3C), NUDT15*3. Additionally, further important PGx markers in genes with known impact on therapeutic outcome have not yet been investigated (Alshabeeb et al., 2022a). This study aimed to investigate the inheritance level of selected variants and star alleles in 15 candidate pharmacogenes for the first time among Saudis. Verification of the mutations in these genes would help in designing a suitable gene panel that outfits the Saudi patients’ need for PGx testing implementation in clinical practice (Alshabeeb et al., 2022b).

## 2 Materials and methods

The DNA of healthy unrelated Saudi participants (n = 95), who voluntarily provided their samples for research purposes and agreed to sign informed consent, was collected from the Biobank at King Abdullah International Medical Research Center (KAIMRC), Ministry of National Guard health Affairs (MNGHA), Riyadh, Saudi Arabia. The total genomic DNA was extracted from whole blood using Puregene Blood Kits (Qiagen, Hilden, Germany, Catalog Number #158389) according to the supplier’s instructions. An automated DNA extraction system (KingFisher™ magnetic system, Thermo Fisher Scientific, Fresno, CA, United States) was used. A DanoDrop (2000/2000c), Thermo Fisher Scientific, Fresno, CA, United States, was used to measure the absorbance of DNA at 260 nm. Working DNA stocks were aliquoted at a concentration of 50 ng/ul and stored at 4 C. The samples were genotyped using the 10x Affymetrix Axiom™ Precision Medicine Diversity Array (PMDA) Plus Kit designed by Thermo Fisher Scientific, United States, (catalog number 951961). The genetic markers to be tested are illustrated in Table 1 and Table 2 as shown in the latest human reference genome version GRCh38.p14. These include *ATIC* (rs4673993), *CACNA1S* (rs772226819), *CFTR* [11 single-nucleotide polymorphisms (SNPs)], *CHRNA5* (rs16969968), *CYP2A6* (6 SNPs), *CYP2B6* (rs3826711), *CYP3A4* (5 SNPs), *DPYD* (rs1801268), *EGFR* (rs121434568), *IFNL3* (rs11881222), *MT-RNR1* (rs267606617, rs267606618, rs267606619), *NAT2* (*14, rs1801279 + rs1208), *RYR1* (rs193922816), *SLC19A1* (rs1051266), and *SLCO1B1* [*9 (rs59502379), *14 (rs11045819 + rs2306283)]. The official full names of the selected pharmacogenes, their ontology, biological function, and gene identification (ID) number are described in the Supplementary Table S1. The given names of the genes match the reported names in the National Center for Biotechnology Information (NCBI) database. The selected markers met two criteria: (1) they showed strong associations with drug response outcome according to well-established PGx databases [levels A–B in the Clinical Pharmacogenetics Implementation Consortium (CPIC), 3–4 in the Dutch Pharmacogenetics Working Group (DPWG), or 1–2 levels in the Pharmacogenomics Knowledgebase (PharmGKB), and (2) their frequency distributions were not previously reported among Saudis. SNPs that showed a genotyping call rate <95% or a Hardy-Weinberg equilibrium (HWE) p-value <0.05 were removed from the study. Linkage disequilibrium was assessed using *r* ^2^ (a squared correlation coefficient). Only markers with *r* ^2^ ≥ 0.6, reflecting moderate-to-strong allelic correlation, were retained for discussion to prioritize robust associations. Allele frequencies of the identified markers in our Saudi cohort were compared with those of other populations (Africans, East Asians, and Europeans) reported in Ensembl and PharmGKB databases (Harrison et al., 2024; Whirl-Carrillo et al., 2021). Fisher’s exact test was used to determine statistically significant differences in allele frequencies between the tested group and other populations. A haplotype analysis software tool version 1.05, prepared by Eliades N-G. and Eliades D. G., was used to determine haplotype frequency of both NAT2*14 and SLCO1B1*14 (Eliades and Eliades, 2009).

**TABLE 1:** Absent pharmacogenetic markers (MAF = 0.0) among the Saudi cohort (n = 95).

Genes	SNPs (n = 26)
*DPYD*	rs1801268
*CACNA1S*	rs772226819
*EGFR*	rs121434568
*CYP3A4*	rs28371759 (*18), rs67666821 (*20)
*CFTR*	rs368505753, rs80282562, rs121908757, rs121909005, rs121909013, rs75527207, rs397508442, rs74503330, rs121909041, rs193922525
*SLCO1B1*	rs59502379 (*9)
*RYR1*	rs193922816
*CYP2A6*	rs568811809 (*20), rs143731390 (*24), rs28399440 (*27), rs8192730 (*28), rs148166815 (*38)
*CYP2B6*	rs3826711
*MT-RNR1*	rs267606617, rs267606618, rs267606619

**TABLE 2:** Detected variants and alleles in nine pharmacogenes and their associated therapeutic agents. Drug/gen associations were derived from PharmGKB and CPIC databases.

Genes (n = 9)	Alleles/SNPs (n = 11)	MAF	Interacting drugs (n = 11)	No. of affected drugs
*ATIC*	rs4673993 (T)	0.71	Methotrexate	1
*CFTR*	rs115545701 (T)	0.005	Ivacaftor	1
*CHRNA5*	rs16969968 (A)	0.35	Nicotine	1
*CYP2A6*	rs1801272 (T, *2)	0.021	Nicotine	
*CYP3A4*	rs2242480 (T)	0.21	Fentanyl	3
	rs35599367 (A, *22)	0.016	Quetiapine	
	rs4986910 (G, *3) rs35599367 (A, *22) rs2242480 (T)	0.005 0.016 0.21	Tacrolimus	
*IFNL3 (IL28B)*	rs11881222 (rs368234815, G)	0.30	Peginterferon Alfa-2a, Peginterferon Alfa-2b, Ribavirin	3
*NAT2*	*14 (rs1801279, A+ rs1208, A)	0.011	Isoniazid	1
*SLC19A1*	rs1051266 (C)	0.48	Methotrexate	1
*SLCO1B1*	*14 (rs11045819, A+ rs2306283, G)	0.14	Rosuvastatin	1

## 3 Results

**FIGURE 1:**
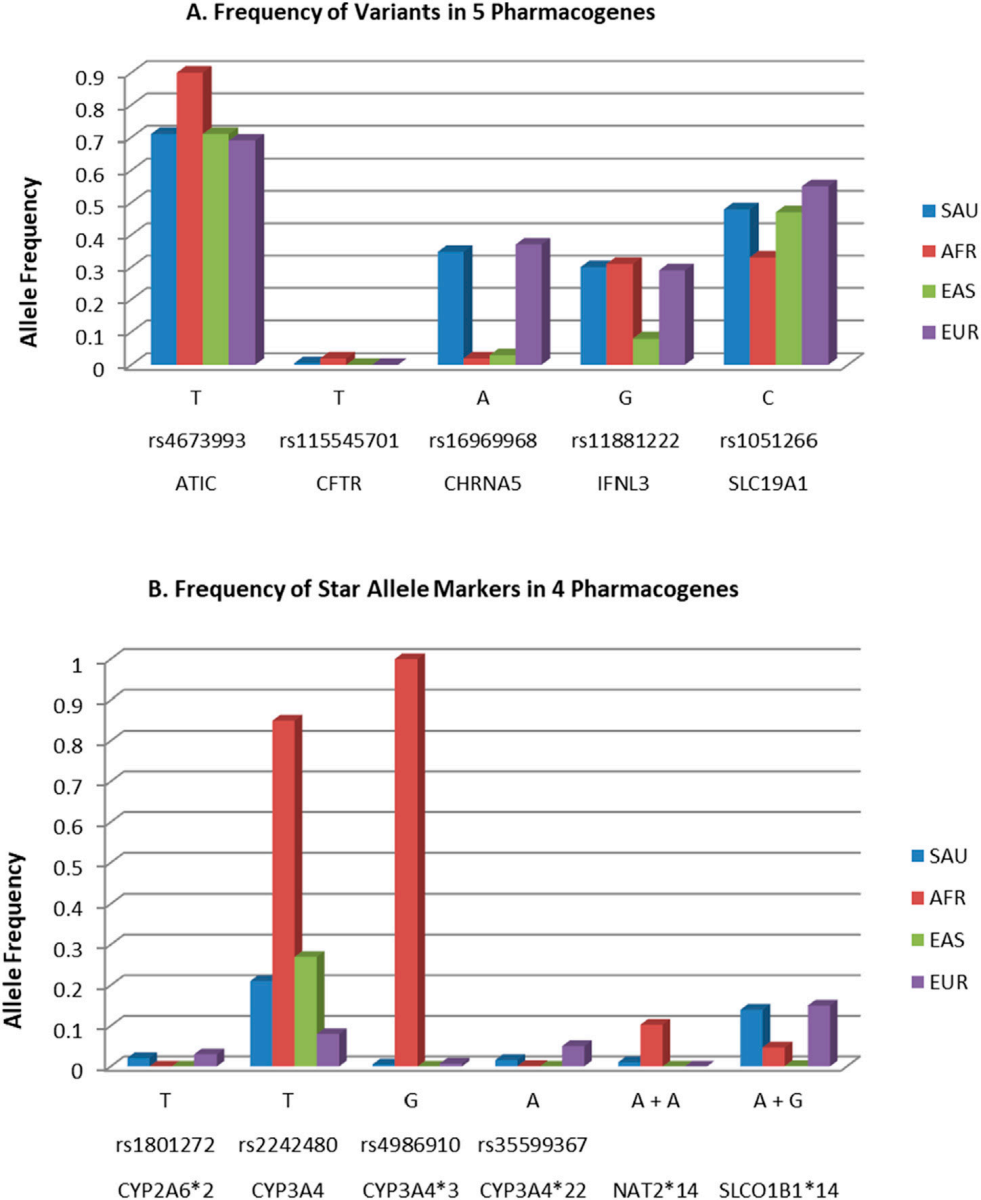
Allele frequency of the detected variants and alleles among Saudis in comparison to previously reported frequencies in Ensembl and PharmGKB databases for African, East Asians, and European populations (Harrison et al., 2024; Whirl-Carrillo et al., 2021). The PGx variants were ploted in **(A)** while the star allele markers are shown in **(B)**. NAT2*14 = (rs1801279, A+ rs1208, A); SLCO1B1*14 = (rs11045819, A+ rs2306283, G).

The enrolled healthy participants were 60 males and 35 females, aged 30.4 ± 7.4 years *versus* 32.3 ± 8.9 years (P-value = 0.87). The average sample genotyping call rate was 99.8%, and none of the samples had a call rate <96.5%. As shown in Table 1, the selected variants in six genes were not detected among the screened individuals [*DPYD *(rs1801268)*, CACNA1S *(rs772226819)*, EGFR* (rs121434568), *RYR1 *(rs193922816)*, CYP2B6 *(rs3826711)*, *and* MT-RNR1* (rs267606617, rs267606618, rs267606619)]. Also, other markers were found to be non-existing in the tested cohort [rs28371759 and rs67666821 in CYP3A4, *10 SNPs (see Table 1) in* CFTR, *rs59502379* *in* SLCO1B1, and 5 alleles (*20, *24, *27, *28, *38) in CYP2A6]. Of the 37 tested markers, 26 were found to be not inherited by the examined participants. In contrast, 11 variants and alleles in nine pharmacogenes were found to be carried by the investigated Saudi cohort. Eight markers were identified in eight different pharmacogenes: ATIC *(rs4673993, minor allele frequency (MAF) = 0.71)*, CFTR *(rs115545701, MAF = 0.005)*, CHRNA5 *(rs16969968, MAF = 0.35)*, CYP2A6 [*2 (rs1801272), MAF = 0.021], IFNL3 *(rs11881222, MAF = 0.30)*, NAT2 [*14 (rs1801279, A+ rs1208, A), MAF = 0.011], SLC19A1 *(rs1051266, MAF = 0.48), and* SLCO1B1 [*14, (rs11045819, A+ rs2306283, G), MAF = 0.14)], Table 2. Whereas, three alleles were detected in CYP3A4 [*3 (rs4986910, MAF = 0.005); rs2242480 (MAF = 0.21); *22 (rs35599367, MAF = 0.016)]. Linkage analysis between rs11881222 and another previously well-studied variant (rs12979860) in *IFNL3* showed a strong correlation (*r* ^2^ = 0.97). Significant differences in allele frequencies were detected for eight variants between the Saudi and African populations, five variants compared to East Asians, and two variants compared to Europeans, as shown in Figure 1 and the Supplementary Table S2. No significant differences were noted for the rare markers rs115545701 in *CFTR *and rs4986910 in* CYP3A4*. The identified mutations in our study cohort have the potential to influence patients’ responses to 11 different therapeutic agents including antimicrobials [n = 4 agents (peginterferon alfa-2a and alfa-2b, and ribavirin affected by *IFNL3*, and isoniazid affected by *NAT2*)], psychiatric [n = 2 agents (quetiapine affected by *CYP3A4*, and nicotine affected by *CHRNA5* and *CYP2A6*)], immunosuppressants/oncology [n = 2 agents (methotrexate affected by *ATIC* and *SLC19A1*, and tacrolimus affected by *CYP3A4*)], analgesic (fentanyl affected by *CYP3A4*), cardiovascular (rosuvastatin affected by *SLCO1B1*), and respiratory (ivacaftor affected by *CFTR*) medications.

## 4 Discussion

This study investigated several variants in 15 genes known to impact patients’ responses to many drugs based on high-quality evidence derived from multiple studies on various populations (Jithesh et al., 2022; Thorn et al., 2013; Alshabeeb et al., 2022a). The selected variants in this study are prevalent in populations with various ancestral backgrounds. However, due to population-specificity and variability of genetic mutations, we intended to identify whether these variants are also common among Saudis. To the best of our knowledge, the tested variants have not yet been screened in the Saudi population, which makes it difficult to design an appropriate gene panel for PGx testing that suits Saudi society’s needs.

The variants in the *DPYD* gene can substantially affect a patient’s response to fluoropyrimidine drugs, potentially leading to severe toxic reactions (Larrue et al., 2024; Da Rocha et al., 2021). Patients carrying certain *DPYD* variants are at an increased risk of experiencing severe toxicity (e.g., gastrointestinal and hematological toxicities) when receiving standard doses of capecitabine or fluorouracil (Wigle et al., 2021). Previously, Goljan et al. reported eight rare variants (rs56038477 was the most frequent mutation, MAF = 0.005) in *DPYD* among the Saudi population (Goljan et al., 2022). One further *DPYD* variant (rs1801268) was tested in the current study, and none of the participants were found to carry the variant allele. According to a previous report (Mizzi et al., 2016), DPYD*13 (rs55886062, c.1679T>G) was also found to be non-inherited by Saudis. We also demonstrated a non-existence of two SNPs: rs121434569 in *EGFR* and rs267606617 in *MT-RNR1*, in a previous study (Alshabeeb et al., 2022b), and similarly, a SNP (rs121434568) in *EGFR* and three (rs267606617, rs267606618, rs267606619) in *MT-RNR1* could not be detected in the current study. These preliminary findings do not confirm the safety or efficacy of drug substrates of *EGFR* (e.g., gefitinib) and *MT-RNR1* (e.g., aminoglycosides) in Saudi populations as a larger sample size cohort need to be tested to replicate our results.

*RYR1* and *CACNA1S* function together to regulate calcium release from calcium channels in the skeletal muscles (Gonsalves et al., 2019). Selected variants in these genes are associated primarily with malignant hyperthermia and muscle disorders when patients are exposed to anesthetic agents and statins (Alvarellos et al., 2016; Sangkuhl et al., 2020; Beam et al., 2017; Haseeb and Thompson, 2021; Isackson et al., 2018). The current Saudi cohort was genotyped for rs772226819 in *CACNA1S* and rs193922816 in *RYR1*, and previously for 20 SNPs in *RYR1* (Alshabeeb et al., 2022b). These variants were absent among the tested Saudi cohort. However, this does not necessarily imply reduced susceptibility to anesthesia-related risks associated with *CACNA1S* and *RYR1* genes among Saudi patients. Further study with a larger sample size is needed to confirm these findings. Another gene is *CYP2B6*, a highly polymorphic cytochrome P450 enzyme that plays a significant role in the drug metabolism of several drugs such as efavirenz and methadone (Desta et al., 2007; Kharasch et al., 2015). A rare variant (rs28399499, MAF = 0.005) in *CYP2B6*, which is known to impact enzyme activity, was reported previously by our team among healthy Saudis (Alshabeeb et al., 2022a). Thus, we subsequently tested another marker (rs3826711) in this study, but it was found to be absent. Further eleven selected variants in *CFTR* commonly associated with cystic fibrosis (CF) in Caucasian and African populations, were explored, and a single marker (rs115545701) was found to be carried by only one participant (MAF = 0.005). These data emphasize the extreme rarity of *CFTR* variants among healthy individuals, but they are common in CF patients. A previous study conducted on 396 CF patients across different regions of Saudi Arabia indicated a carriage of different ten *CFTR* variants (Banjar et al., 2021). Ivacaftor is a novel drug designed to treat cystic fibrosis, targeting only those who carry disease-causative *CFTR* mutations (Anderson et al., 2023).

Carriage of ten other mutations in nine genes was confirmed in the tested Saudi participants. The mutations have the potential to affect the response of patients to 10 drugs commonly used in the country’s healthcare system. Two variants in *ATIC* (rs4673993, T allele) and *SLC19A1* (rs1051266, C allele), which impact methotrexate (MTX) efficacy in rheumatoid arthritis (Chen et al., 2017), demonstrated high prevalence among the enrolled Saudis with allele frequencies of 71% and 48%, respectively. These high-frequency distributions within our population necessitate prior genetic testing for the variants preemptively for each patient predicted to be exposed to MTX. Carriage of these variants is associated with decreased treatment efficacy (Lima et al., 2016). Thus, higher doses of MTX might be necessary to achieve satisfactory therapeutic outcome. Clinicians may also consider using alternative or additional disease-modifying antirheumatic drugs to improve treatment results (Qiu et al., 2017). Additionally, two markers in *CHRNA5* (rs16969968) and *CYP2A6* (rs1801272) genes, associated with increased risk for nicotine dependence in smokers (Lee et al., 2018; Pianezza et al., 1998), were proven to be carried by the Saudis involved in our study. These mutations have significant implications for smoking behavior and cessation. Another mutation in our population is NAT2*14, a slow acetylator SNP which affects isoniazid (antituberculosis) metabolism. This allele was reported with an increased risk of isoniazid-induced liver injury (McDonagh et al., 2014).

In this study, a large portion of the Saudi participants (30%) was found to be carriers of the *IFNL3* variant (rs11881222). This mutation is associated with poorer antiviral response in hepatitis C or human immunodeficiency virus (HIV) disease management (Chen et al., 2011). However, we noticed a strong LD (*r* ^2^ = 0.97) between rs11881222 and a previously reported *IFNL* SNP (rs12979860) (Al-Qahtani et al., 2015). This linkage was also indicated in East Asians and Europeans (*r* ^2^ = 0.95 and 0.91, respectively) (Prokunina-Olsson, 2019). *CYP3A4* is a crucial enzyme in drug metabolism, responsible for metabolizing approximately 50%–60% of clinically prescribed drugs. Thus, it has significant implications for personalized medicine and drug therapy optimization (Zhang et al., 2024). Two markers in *CYP3A4* (−290A < G and 902 T > C, predicted to impact the metabolism of nifedipine and vitamin D, respectively) were previously reported among Saudis (Tayeb et al., 2000; Al-Ashwal et al., 2023). Further, our study identified two rare (*3 and *22) and one common (rs2242480) *CYP3A4* alleles. These alleles have the potential to impact tacrolimus metabolism and affect its blood concentrations (Hesselink et al., 2003; Oetting et al., 2018). In addition, increased exposure to quetiapine (an antipsychotic) and fentanyl (an opioid painkiller) was reported in patients carrying CYP3A4*22 and rs2242480, respectively (Mulder et al., 2021; Zhang et al., 2018).

Furthermore, statin-induced myopathy and rhabdomyolysis in association with various markers in *SLCO1B1* were extensively studied (Vladutiu and Isackson, 2009). Previous reports confirmed the existence of SLCO1B1*5 among Saudis (Mizzi et al., 2016; Alshabeeb et al., 2023). This study also detected the SLCO1B1*14 genetic allele in 14% of the tested Saudi cohort. This marker is associated with increased OATP1B1 transporter expression and activity causing profound loss of rosuvastatin efficacy (Ho et al., 2006). SLCO1B1*14 is a haplotype mutation comprised of two variants: rs11045819 and rs2306283 variants. The variant rs11045819 was independently found to be associated with decreased exposure to simvastatin and rifampin in single reports that need further replication (Mykkänen et al., 2022; Kwara et al., 2014).

These data shed light on the missing heritability related to PGx markers across our underrepresented Saudi population, though further studies are needed to screen larger sample size. Identifying the level of distribution of genetic factors that influence drug safety and efficacy can lead to more informed treatment decisions (Chenchula et al., 2024). The distinct genetic makeup of the Saudi population, shaped by unique geographic and demographic factors (Khubrani, 2020; Aleissa et al., 2022), necessitates studies on their drug response variability. Our study confirmed the uniqueness of the Saudi genome, as nine out of the 11 identified markers showed statistically significant differences in their allele frequencies between the Saudi group and at least one of the populations with different ancestral backgrounds. The variant selection in this study was informed by prior findings from Caucasian population studies, necessitating complementary next-generation sequencing (NGS) approaches [whole-genome sequencing, exome sequencing, or targeted sequencing of regions near actionable SNPs] to fully characterize Saudi-specific genetic associations. While the Axiom PMD Research Array provided cost-effective detection of shared variants between Saudis and other populations, three key limitations emerged: 1) its coverage is limited to pre-designed probes (fails to detect novel variants), 2) covers <5% of regulatory regions, and 3) unable to detect structural variants larger than 50 kilobases (kb)] (Wheelan et al., 2008; Miller and Tang, 2009).

## 5 Conclusion

Eleven variants in nine genes, with the potential to impact 11 therapeutic agents, were found to be inherited among the enrolled Saudi cohort. The identified data can be integrated into clinical practice in Saudi Arabia. This includes developing guidelines for healthcare providers on using genetic information to tailor drug therapy.

## Data Availability

The datasets presented in this study can be found in online repositories. The names of the repository/repositories and accession number(s) can be found in the article/Supplementary Material.
