# Supplementary material for: Unveiling the heritability of selected unexplored pharmacogenetic markers in the Saudi population
Source: Front Pharmacol. 2025 May 1;16:1559399. doi: 10.3389/fphar.2025.1559399 (PMC12078325; doi:10.3389/fphar.2025.1559399)
Supplement: Supplementary file 1 [file Table1.docx]

**Supplementary Table S1:** Official full names of pharmacogenes, ontology, biological function, and gene ID as seen in the National Center for Biotechnology Information (NCBI) database.

| Symbol | Gene Name | Gene Ontology | Biological function | Gene ID as seen in NCBI |
| --- | --- | --- | --- | --- |
| ATIC | 5-aminoimidazole-4-carboxamide ribonucleotide formyltransferase/IMP cyclohydrolase | Cytoplasm | Enzyme | 471 |
| CACNA1S | Calcium voltage-gated channel subunit alpha1 S | Plasma Membrane | Ion channel | 779 |
| CFTR | CF transmembrane conductance regulator | Plasma Membrane | Ion channel | 1080 |
| CHRNA5 | Cholinergic receptor nicotinic alpha 5 subunit | Plasma Membrane | Receptor | 1138 |
| CYP2A6 | Cytochrome P450 family 2 subfamily A member 6 | Cytoplasm | Enzyme | 1548 |
| CYP2B6 | Cytochrome P450 family 2 subfamily B member 6 | Cytoplasm | Enzyme | 1555 |
| CYP3A4 | Cytochrome P450 family 3 subfamily A member 4 | Cytoplasm | Enzyme | 1576 |
| DPYD | dihydropyrimidine dehydrogenase | Cytoplasm | Enzyme | 1806 |
| EGFR | Epidermal growth factor receptor | Plasma Membrane | Receptor | 1956 |
| IFNL3 (IL28B) | Interferon lambda 3 | Extracellular Space | Cytokine | 282617 |
| MT-RNR1 | Mitochondrially encoded 12S RNA | Cytoplasm | Insulin sensitivity regulator | 4549 |
| NAT2 | N-acetyltransferase 2 | Cytoplasm | Enzyme | 10 |
| RYR1 | Ryanodine receptor 1 | Cytoplasm | Ion channel | 6261 |
| SCN1A | Sodium voltage-gated channel alpha subunit 1 | Plasma Membrane | Ion channel | 6323 |
| SLC19A1 | Solute carrier family 19 member 1 | Plasma Membrane | Transporter | 6573 |
| SLCO1B1 | Solute carrier organic anion transporter family member 1B1 | Plasma Membrane | Transporter | 10599 |
