# Supplementary material for: Unveiling the heritability of selected unexplored pharmacogenetic markers in the Saudi population
Source: Front Pharmacol. 2025 May 1;16:1559399. doi: 10.3389/fphar.2025.1559399 (PMC12078325; doi:10.3389/fphar.2025.1559399)
Supplement: Supplementary file 2 [file Table2.docx]

**Supplementary Table S2:** Allele frequency comparison of the newly explored PGx markers between the Saudi cohort and other populations.

| Gene/Allele | SNP | Variant | SAU (n=95) | AFR (n=661) | EAS (n=504) | EUR (n=503) |
| --- | --- | --- | --- | --- | --- | --- |
|  |  |  | Minimum Allele Frequency (MAF) | | | |
| ATIC | rs4673993 | T | 0.71 | 0.9 | 0.71 | 0.69 |
| *P-value* |  | | | **< 0.0001** | 0.84 | 0.84 |
| CFTR | rs115545701 | T | 0.005 | 0.02 | 0 | 0 |
| *P-value* |  | | | 0.24 | 0.16 | 0.16 |
| CHRNA5 | rs16969968 | A | 0.35 | 0.02 | 0.03 | 0.37 |
| *P-value* |  | | | **< 0.0001** | **< 0.0001** | 0.68 |
| CYP2A6*2 | rs1801272 | T | 0.021 | 0 | 0 | 0.03 |
| *P-value* |  | | | **0.0002** | **0.0006** | 0.64 |
| CYP3A4 | rs2242480 | T | 0.21 | 0.85 | 0.27 | 0.08 |
| *P-value* |  |  |  | **< 0.0001** | 0.10 | **< 0.0001** |
| CYP3A4*3 | rs4986910 | G | 0.005 | 0.001 | 0 | 0.007 |
|  |  |  |  | 0.24 | 0.16 | 1.0 |
| CYP3A4*22 | rs35599367 | A | 0.016 | 0.001 | 0 | 0.05 |
|  |  |  |  | **0.007** | **0.004** | **0.03** |
| IFNL3 | rs11881222 | G | 0.30 | 0.31 | 0.08 | 0.29 |
| *P-value* |  |  |  | 0.80 | **< 0.0001** | 0.79 |
| NAT2*14 |  | A + A | 0.011 | 0.103 | 0 | 0 |
| *P-value* |  |  |  | **< 0.0001** | 0.16 | 0.16 |
| SLC19A1 | rs1051266 | C | 0.48 | 0.33 | 0.47 | 0.55 |
| *P-value* |  |  |  | **< 0.0001** | 0.81 | 0.08 |
| SLCO1B1*14 |  | A + G | 0.14 | 0.047 | 0.0014 | 0.15 |
| *P-value* |  |  |  | **0.0016** | **< 0.0001** | 0.87 |

Saudis: SAU, African: AFR, East Asians: EAS, and Europeans: EUR
